# Beyond Glycemia: Pharmacology-Driven Ketogenesis and Euglycemic DKA with SGLT2 Inhibitors—A Practical Review for Acute Care

**DOI:** 10.3390/jpm16030156

**Published:** 2026-03-10

**Authors:** Massimo Meco, Emiliano Agosteo, Pierluigi Zulli, Fulvio Nisi, Enrico Giustiniano

**Affiliations:** 1 Department of Anaesthesia and Intensive Care, San Carlo Clinic, 20037 Paderno Dugnano, Italy; emiliano.agosteo@clinicasancarlo.it; 2 Department of Anaesthesia and intensive Care, European Hospital, 00149 Rome, Italy; pierluigi.zulli@europeanhospital.it; 3 Department of Anesthesia and Intensive Care, IRCCS Humanitas Research Hospital, 20089 Rozzano, Italy; fulvio.nisi@humanitas.it; 4 Department of Biomedical Sciences, Humanitas University, 20072 Pieve Emanuele, Italy; enrico.giustiniano@humanitas.it

**Keywords:** SGLT2 inhibitors, euglycemic diabetic ketoacidosis, beta-hydroxybutyrate, high anion gap metabolic acidosis, emergency department, perioperative management, sick-day rules

## Abstract

Sodium–glucose cotransporter-2 inhibitors (SGLT2i) are widely prescribed for type 2 diabetes, heart failure, and chronic kidney disease. A rare but dangerous adverse event is SGLT2i-associated diabetic ketoacidosis, often euglycaemic (euDKA), and therefore easy to miss. This narrative review summarizes mechanisms, triggers (fasting, dehydration, infection, perioperative stress), and ketone-centred pathways for diagnosis, treatment, and prevention in acute-care settings. Management priorities are volume resuscitation, blood β-hydroxybutyrate and acid–base testing, insulin infusion with dextrose to suppress ketogenesis, and electrolyte replacement. Prevention relies on “sick-day” rules, perioperative drug withholding, and a low threshold for ketone testing in any patient with high-anion-gap acidosis, despite normal or mildly elevated glucose.

## 1. Introduction

Sodium–glucose cotransporter 2 inhibitors (SGLT2i) have rapidly evolved from glucose-lowering drugs into cross-disciplinary cardiometabolic therapies prescribed for cardiovascular and renal risk reduction in type 2 diabetes mellitus (T2DM). Landmark outcome trials with empagliflozin and canagliflozin established benefits on major cardiovascular and renal endpoints and decisively broadened the clinical rationale for using this drug class beyond glycemic targets [1,2]. Importantly, the modern “use-case” of SGLT2i is no longer confined to diabetology: patients with multiple comorbidities (heart failure, chronic kidney disease, frailty, recurrent hospitalizations) are increasingly exposed to SGLT2i in real-world practice, meaning that emergency physicians, internists, intensivists, anesthesiologists, and perioperative teams now encounter SGLT2i-treated patients routinely—often outside specialist diabetes settings.

The appeal of SGLT2i stems from a kidney-centered mechanism that triggers systemic adaptations. By inhibiting proximal tubular glucose reabsorption, these agents induce insulin-independent glycosuria, coupled to natriuresis and osmotic diuresis, thereby influencing plasma volume status, blood pressure, and intraglomerular hemodynamics—effects widely considered central to nephroprotection and heart failure benefit [3]. Beyond hemodynamics, SGLT2i reprogram endocrine–substrate signaling: they reduce insulin requirements and shift the insulin-to-glucagon balance, promoting lipolysis and increasing hepatic ketone production [3]. This “substrate shift” has been proposed as a contributor to cardiovascular benefit, encapsulated by the “thrifty substrate” hypothesis, where mildly increased circulating ketones might support myocardial energetics under chronic stress conditions [4]. In most patients, this metabolic remodeling remains subclinical and potentially favorable. However, it also provides the mechanistic foundation for the most clinically consequential acute adverse event linked to SGLT2i—diabetic ketoacidosis (DKA), and in particular the euglycemic phenotype (euDKA).

Euglycemic DKA has emerged as an especially challenging entity because it breaks the cognitive shortcut that many acute-care clinicians still apply: “no marked hyperglycemia, no DKA.” In euDKA, patients can present with severe ketonemia and high anion-gap metabolic acidosis despite normal or only mildly elevated glucose, leading to diagnostic delay when hyperglycemia is implicitly treated as a prerequisite [5,6,7]. This is not merely semantic. DKA is fundamentally a ketone-driven acid–base emergency, and the pathophysiology of euDKA underscores that glucose concentration can become a misleading signal when glycosuria actively removes glucose from the circulation. SGLT2i can therefore “mask” the classic hyperglycemic alarm, while ketogenesis accelerates in the background—particularly when catabolic stressors superimpose on the pharmacodynamic milieu created by SGLT2i.

The clinical relevance of euDKA is amplified in precisely those contexts that are common in emergency departments and perioperative medicine: reduced oral intake or fasting, dehydration, vomiting, intercurrent infection, surgery and procedural stress, and reductions/omission of insulin therapy [5,6,7]. In these scenarios, SGLT2i-related glycosuria may maintain deceptively normal glucose values, while relative insulin deficiency and counter-regulatory hormone excess intensify lipolysis and ketone production. The resulting presentation can be nonspecific—nausea, abdominal pain, dyspnea/tachypnea, malaise—readily misattributed to gastroenteritis, postoperative ileus, sepsis, pulmonary pathology, anxiety-related hyperventilation, or medication intolerance. The consequence is a “diagnostic trap”: the patient appears clinically unwell, yet point-of-care glucose is not striking, and ketoacidosis may not enter the initial differential. Once unrecognized, euDKA can progress rapidly and require ICU-level care, even though timely ketone testing and protocolized management can be lifesaving.

Regulatory agencies and real-world analyses have reinforced euDKA as an uncommon but high-impact safety signal. Safety communications highlighted that ketoacidosis can occur in SGLT2i-treated patients with glucose that is not markedly elevated, explicitly warning clinicians not to exclude DKA on glycemia alone [8]. Observational data further supported a signal of increased DKA risk after initiation of SGLT2i compared with some alternative glucose-lowering agents, underscoring the importance of context, patient selection, and trigger recognition [9]. Expert reviews and consensus recommendations have therefore emphasized risk mitigation, early recognition, and a ketone-driven diagnostic approach in acute care settings [10].

**Figure 1:**
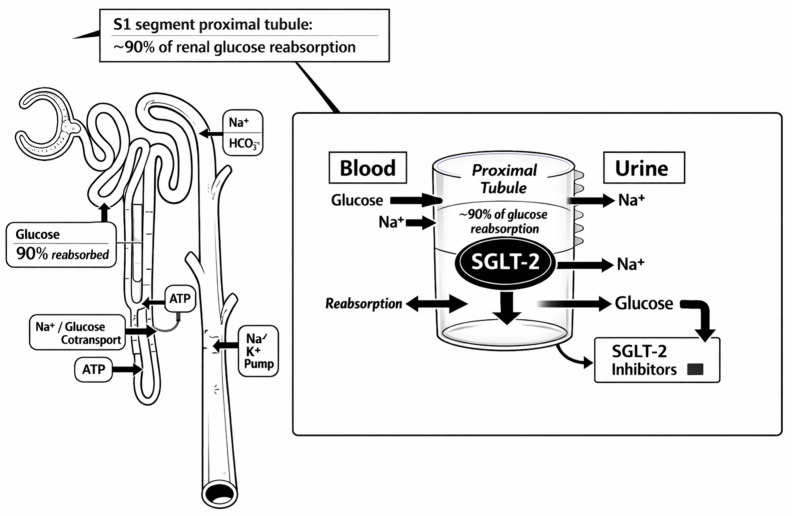
Renal effects of SGLT2 inhibition. SGLT2 inhibition in the proximal renal tubule induces insulin-independent glycosuria and natriuresis, leading to osmotic diuresis, mild plasma volume contraction, and restoration of tubuloglomerular feedback, mechanisms central to cardiorenal protection. At the endocrine–metabolic level, reduced glucose availability lowers insulin secretion and increases relative glucagon signaling, shifting substrate utilization toward lipolysis and hepatic ketogenesis. While this adaptive substrate shift may contribute to cardiovascular benefit under stable conditions, superimposed stressors (fasting, dehydration, infection, surgery, or insulin reduction) can accelerate ketone production while glycosuria blunts hyperglycemic warning signs, thereby predisposing susceptible patients to euglycemic DKA.

**Figure 2:**
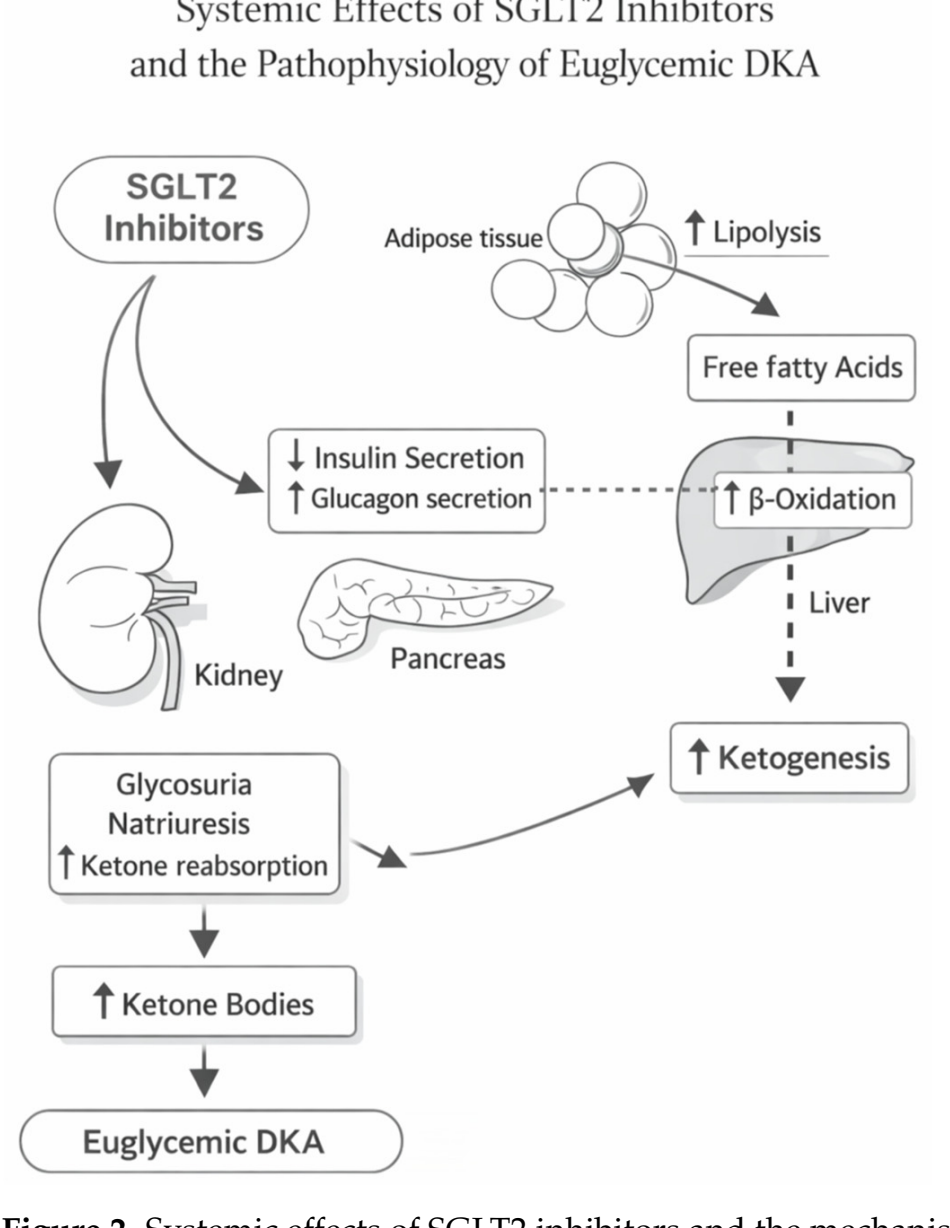
Systemic effects of SGLT2 inhibitors and the mechanistic pathway to euglycemic diabetic ketoacidosis (euDKA). SGLT2 inhibition promotes renal glycosuria and natriuresis with osmotic diuresis and, in susceptible contexts, increased renal ketone reabsorption. Concomitantly, reduced insulin secretion and relative increases in glucagon signaling favor adipose lipolysis, raising circulating free fatty acids. Hepatic β-oxidation of free fatty acids drives ketogenesis, leading to the accumulation of ketone bodies and the development of euDKA despite absent or only mild hyperglycemia. The schematic highlights how SGLT2 inhibitor–related glucose loss can blunt hyperglycemic warning signs while ketone production accelerates during catabolic stress.

Against this background, the objective of this narrative review is to provide a mechanism-to-bedside framework tailored to emergency and perioperative clinicians who increasingly manage SGLT2i-exposed patients. We first summarize the pharmacology and pharmacodynamics of SGLT2i relevant to euDKA risk, including the renal mechanism and systemic metabolic adaptations (Figure 1). We then integrate current mechanistic understanding of euDKA, highlighting how SGLT2i lowers the threshold for ketogenesis and decouples ketone severity from glucose levels (Figure 2). Finally, we translate these mechanisms into a pragmatic acute-care workflow: when to suspect euDKA, which laboratories should be prioritized, how to distinguish euDKA from classic DKA and other causes of high anion-gap metabolic acidosis, and how to treat euDKA safely with insulin plus early dextrose, fluids, and meticulous electrolyte management—while simultaneously addressing triggers and implementing prevention strategies that preserve the substantial benefits of SGLT2i therapy.

## 2. Methods

### 2.1. Search Strategy and Selection Criteria

This narrative review aims to translate the pharmacology of sodium–glucose cotransporter 2 inhibitors (SGLT2i) into a pragmatic acute-care framework for the recognition, diagnosis, management, and prevention of euglycemic diabetic ketoacidosis (euDKA). To enhance transparency and reduce selection bias, we conducted a structured literature search complemented by targeted manual searches of reference lists, clinical guidelines, and regulatory safety communications.

Databases and search strategy. We searched PubMed/MEDLINE, Scopus, and Web of Science from database inception to 31 January 2026 (last search: 31 January 2026). Searches combined controlled vocabulary (where applicable) and free-text terms related to SGLT2 inhibitors, euDKA, and acute-care contexts. Core terms included *“SGLT2 inhibitor*” OR “sodium–glucose cotransporter 2 inhibitor*” OR dapagliflozin OR empagliflozin OR canagliflozin OR ertugliflozin* AND *“euglycemic diabetic ketoacidosis” OR “euglycaemic DKA” OR “diabetic ketoacidosis” OR “ketosis” OR “beta-hydroxybutyrate” OR “anion gap metabolic acidosis”*. Additional terms captured high-risk settings and workflows (*perioperative*, *surgery*, *fasting*, *infection*, *critical care*, *emergency department*). Reference lists of relevant reviews, consensus statements, and seminal reports were screened (snowballing) to identify additional pertinent publications.

### 2.2. Example PubMed Search String (Used as a Backbone)

((“SGLT2 inhibitor*” OR “sodium-glucose cotransporter 2 inhibitor*” OR dapagliflozin OR empagliflozin OR canagliflozin OR ertugliflozin) AND (“euglycemic diabetic ketoacidosis” OR “euglycaemic DKA” OR “diabetic ketoacidosis” OR ketosis OR “beta-hydroxybutyrate” OR “anion gap metabolic acidosis”)) AND (perioperative OR surgery OR fasting OR infection OR “emergency department” OR “critical care”).

Eligibility and evidence prioritization. We considered clinical trials, observational and population-based studies, pharmacovigilance analyses, case series/reports (to characterize presentation patterns and triggers), mechanistic/physiological studies, consensus statements and guidelines, and regulatory safety communications. Evidence was prioritized if it: (i) addressed euDKA in the setting of SGLT2i exposure; (ii) provided actionable information for emergency, perioperative, or critical care practice (diagnosis, treatment protocols, prevention); or (iii) clarified mechanistic links between SGLT2i pharmacodynamics (glycosuria, insulin-to-glucagon shift, ketone metabolism, volume status) and ketogenesis under stress.

Selection and synthesis. Titles and abstracts were screened for relevance to euDKA mechanisms and acute-care workflows; full texts were reviewed when they contributed materially to one or more sections of the manuscript. Evidence was synthesized thematically (pharmacology/pharmacodynamics; pathophysiology; epidemiology; triggers and risk phenotypes; ED diagnosis; protocolized management; perioperative prevention; special populations), emphasizing consistency across sources and clinically implementable recommendations. As a narrative review, no PRISMA-guided systematic appraisal or quantitative meta-analysis was performed; however, when available, higher-level evidence (guidelines/consensus statements, large trials/program data, and population-based analyses) was preferentially cited, with case-based literature used primarily to illustrate diagnostic pitfalls and acute-care presentations.

## 3. Pharmacology and Pharmacodynamics of SGLT2 Inhibitors

### 3.1. Molecular Target and Renal Glucose Handling

Under physiologic conditions, virtually all filtered glucose is reclaimed along the proximal tubule, with SGLT2 in the early proximal segment accounting for the majority of reabsorption and SGLT1 in the more distal segment providing “back-up” reabsorption for the remainder [11]. SGLT2 inhibitors reduce proximal tubular glucose reabsorption and thereby increase urinary glucose excretion, producing an insulin-independent glucose-lowering effect that is fundamentally “kidney-facing” rather than pancreas-facing [12]. Importantly, the magnitude of glycosuria depends not only on systemic exposure but also on filtered drug delivery to the tubular lumen and on the prevailing filtered glucose load; as a result, the pharmacologic effect can be sustained and clinically relevant beyond a single dosing interval. From a practical perspective, this mechanism means that patients may remain in a state of active glucosuria—and therefore in a state of reduced circulating glucose “signal”—even when the last dose is not recent, particularly during acute illness, altered intake, or perioperative stress (Figure 1).

### 3.2. Pharmacodynamics: Hemodynamic and Metabolic Effects as a Continuum

The pharmacodynamic profile of SGLT2 inhibition extends beyond glycosuria and is best viewed as a continuum of renal, hemodynamic, and endocrine–substrate adaptations. At the renal–hemodynamic level, glycosuria is coupled to natriuresis and osmotic diuresis, producing modest plasma volume contraction and reductions in blood pressure while restoring tubuloglomerular feedback and lowering intraglomerular pressure—effects central to nephroprotection [12]. These changes are typically beneficial in chronic cardiorenal disease; however, they also create a physiologic “tilt” toward volume depletion when superimposed stressors occur (vomiting, reduced intake, fever, perioperative fasting), a point that becomes highly relevant to euDKA because dehydration can impair renal clearance of ketones and hydrogen ions, amplifying the acid–base disturbance (Table 1).

At the endocrine–metabolic level, SGLT2i shift the hormonal milieu toward a more ketone-permissive state. By lowering plasma glucose through urinary losses, they reduce endogenous insulin secretion and/or reduce exogenous insulin requirements; simultaneously, they are associated with increased glucagon signaling, lowering the insulin-to-glucagon ratio and favoring adipose lipolysis and hepatic ketogenesis [13]. Experimental work supports a direct pancreatic alpha-cell component, with SGLT2 inhibition linked to increased glucagon secretion [13]. Clinically, small but consistent rises in circulating ketone bodies are frequently observed during SGLT2i therapy and have been proposed as part of the “thrifty substrate” hypothesis—i.e., a shift toward ketone utilization that could support myocardial energetics and contribute to cardiovascular benefit in chronic disease. The key point for acute care is that the same adaptive metabolic shift becomes maladaptive when combined with acute reductions in carbohydrate availability, abrupt insulin dose reduction/omission, or counter-regulatory hormone surges: under those conditions, lipolysis and hepatic ketogenesis can accelerate while the expected hyperglycemic “alarm” is attenuated.

### 3.3. Pharmacokinetics and Clinically Relevant Persistence: Why Timing Alone Is Misleading

SGLT2 inhibitors are administered once daily, and their elimination half-lives support this regimen; yet the clinically important issue in acute care is not half-life per se, but pharmacodynamic persistence—ongoing glycosuria and the associated substrate/hormonal milieu [14]. Multiple factors can extend the “clinical footprint” of SGLT2i beyond the last dose: continued luminal exposure within the nephron, reduced carbohydrate intake, perioperative stress, and dehydration. Consequently, recent exposure should be considered relevant even if the last tablet was taken several days earlier, particularly around surgery or intercurrent illness [14]. In practical terms, when an SGLT2i-exposed patient presents with unexplained nausea, tachypnea, or high anion-gap acidosis, clinicians should not dismiss the drug’s contribution solely because the reported last dose was “not today.” (Table 2).

### 3.4. Drug–Context Interactions: The Pharmacodynamic Triad That Enables euDKA

EuDKA should be conceptualized as a context-dependent metabolic decompensation in which SGLT2i pharmacology lowers the threshold for ketogenesis under stress. Three converging pharmacodynamic forces are particularly important: (i) glycosuria that blunts or masks hyperglycemia, thereby removing the classical diagnostic cue; (ii) reduced insulin tone with relative glucagon predominance, which drives lipolysis and hepatic beta-oxidation; and (iii) enhanced ketone production and accumulation, especially when dehydration limits renal clearance [15]. In this setting, glucose concentration becomes an unreliable proxy for severity: ketonemia and acidosis can worsen despite only mild elevations in plasma glucose. Regulatory safety communications have emphasized exactly this point—ketoacidosis may occur in SGLT2i-treated patients without markedly elevated glucose—highlighting the need for ketone- and acid–base–driven diagnostic strategies in emergency and perioperative workflows. Observational analyses have also supported an increased risk of DKA after SGLT2i initiation compared with some alternative glucose-lowering therapies, underscoring that patient selection and triggers (fasting, infection, insulin reduction, surgery) materially influence risk. Taken together, euDKA is best understood not as a paradox, but as the predictable intersection between SGLT2i-induced glycosuria, a ketone-favoring endocrine state, and superimposed catabolic stress—precisely the combination often encountered in acute care settings (Figure 2).

## 4. Pathophysiology of DKA and euDKA in SGLT2i Users

**Figure 3:**
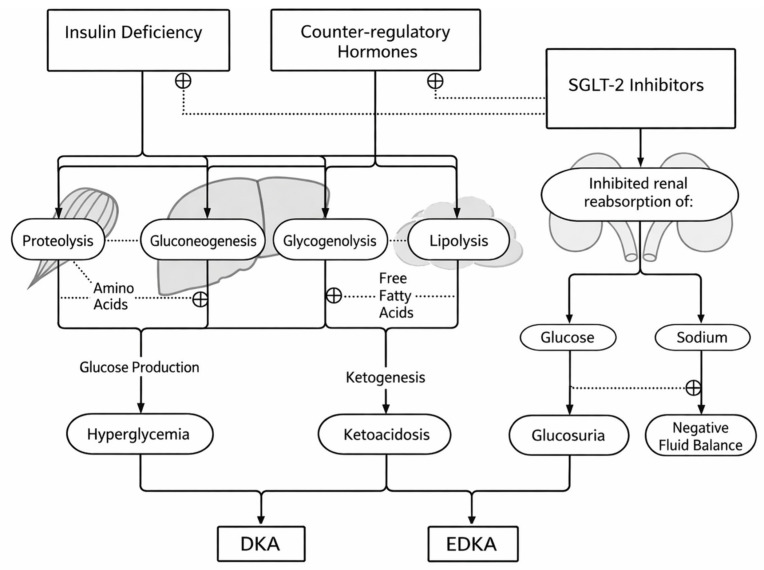
Pathophysiological pathways leading to classic diabetic ketoacidosis (DKA) and euglycemic DKA (euDKA) in patients exposed to SGLT2 inhibitors. Insulin deficiency and counter-regulatory hormone excess drive proteolysis, gluconeogenesis/glycogenolysis, and lipolysis, increasing hepatic fatty acid oxidation and ketogenesis, resulting in ketoacidosis and (in classic DKA) marked hyperglycemia. In SGLT2 inhibitor–exposed patients, inhibited renal glucose and sodium reabsorption promotes glucosuria and natriuresis with negative fluid balance, which can blunt hyperglycemia while sustaining the ketone-driven acid–base disturbance, thereby favoring an euglycemic presentation (euDKA). The schematic emphasizes the decoupling between ketone burden and plasma glucose under SGLT2 inhibition and the convergent role of dehydration and catabolic stress in precipitating both phenotypes.

Classical diabetic ketoacidosis (DKA) represents the final common pathway of absolute or relative insulin deficiency combined with excess counter-regulatory hormones (glucagon, catecholamines, cortisol, and growth hormone). This hormonal milieu simultaneously increases hepatic glucose output (via gluconeogenesis and glycogenolysis) and, crucially, shifts adipose and hepatic metabolism toward accelerated lipolysis and hepatic mitochondrial beta-oxidation of free fatty acids, leading to overproduction of ketone bodies (predominantly β-hydroxybutyrate) and the development of high anion-gap metabolic acidosis [16,17]. In other words, DKA is fundamentally a ketone-driven acid–base emergency, with hyperglycemia representing an associated—often prominent—feature rather than the defining pathophysiologic core [16,17]. In euglycemic DKA (euDKA), the same ketone-centric process unfolds, but the typical glycemic signature is altered. In patients exposed to SGLT2 inhibitors, ongoing urinary glucose losses lower circulating glucose and blunt the hyperglycemic “alarm”, such that substantial ketonemia and severe acidosis can develop despite normal or only mildly elevated plasma glucose. This decoupling between glucose and ketone burden is the pathophysiologic hallmark of euDKA and the key reason diagnosis is often delayed when clinicians anchor on glycemia rather than on acid–base status and ketones. The renal and volume components are not secondary details: they strongly modulate both severity and tempo. SGLT2i-induced glycosuria is accompanied by osmotic diuresis and natriuresis, predisposing to volume depletion, particularly when compounded by reduced intake, vomiting, fever, or perioperative fasting. Hypovolemia then amplifies euDKA through at least two synergistic mechanisms. First, reduced effective circulating volume lowers renal perfusion and can decrease glomerular filtration, impairing renal clearance of ketones and hydrogen ions and thereby worsening acidemia [17]. Second, hypovolemia and stress stimulate counter-regulatory hormone release, which further lowers the insulin-to-glucagon ratio and accelerates lipolysis and hepatic ketogenesis—creating a self-reinforcing spiral in which ketone production increases while ketone clearance decreases [17]. As a result, euDKA commonly requires a precipitating stressor (infection, fasting, surgery, insulin dose reduction), not because SGLT2i “cause” DKA in isolation, but because they lower the threshold at which a catabolic stress state tips into clinically significant ketoacidosis. These integrated mechanisms have direct therapeutic implications. Because dehydration worsens both acidemia and ketone accumulation, early isotonic fluid resuscitation is not merely supportive; it is a pathophysiology-targeted intervention that restores renal perfusion, improves ketone and acid clearance, and helps reverse the counter-regulatory surge that perpetuates ketogenesis. In parallel, insulin therapy is required to suppress lipolysis and hepatic ketone production [16,17]. In euDKA, however, insulin administration often demands earlier concomitant dextrose to avoid hypoglycemia while maintaining sufficient insulin exposure to terminate ketogenesis—an operational consequence of the same glycosuria-driven decoupling that underlies the condition (Figure 3).

## 5. Epidemiology and Magnitude of Risk

Across large cardiovascular outcome programs, diabetic ketoacidosis (DKA) events remain uncommon, and this low absolute incidence is an important part of the overall risk–benefit profile that supports the widespread use of SGLT2 inhibitors (SGLT2i). Nevertheless, because DKA is a high-acuity event with potential need for ICU-level care, even rare occurrences have outsized clinical relevance—especially when presentation is atypical and diagnosis can be delayed. Post-marketing surveillance and pharmacovigilance reports were pivotal in identifying and consolidating the signal that SGLT2i are associated with DKA, including euglycemic DKA, and in emphasizing that glucose may not be markedly elevated in affected patients. In practical terms, this safety signal reshaped the way acute-care clinicians should interpret metabolic acidosis in SGLT2i-exposed individuals: the absence of severe hyperglycemia does not meaningfully reduce the need to consider ketoacidosis.

Trial-level and programmatic data support the same message. Within the canagliflozin type 2 diabetes clinical program, serious DKA-related events were reported and characterized as rare but clinically important, helping define typical precipitating contexts and reinforcing the role of stressors and insulin deficiency states in event generation [18]. These observations are consistent with a “threshold model” of euDKA: SGLT2i do not usually produce ketoacidosis in isolation, but they lower the threshold at which catabolic stress, reduced carbohydrate availability, or reduced insulin exposure precipitates clinically overt ketoacidosis.

Real-world studies have complemented trial observations by examining risk under routine prescribing patterns, where heterogeneity in patient selection, comorbid burden, and trigger exposure is greater. Population-level analyses have suggested that SGLT2i initiation is associated with a higher risk of DKA compared with some alternative glucose-lowering therapies, even though the absolute risk remains low. Importantly, this relative-risk framing can be misleading at the bedside if interpreted without context: the clinically useful message is not that euDKA is “common,” but that it is predictably enriched in specific settings—recent initiation, intercurrent illness, reduced intake, perioperative fasting, insulin dose reduction/omission, dehydration, and possibly limited insulin reserve. In other words, event rates are highly context-dependent, and the presence of triggers often matters more than background incidence estimates.

For emergency and perioperative clinicians, the epidemiology therefore translates into a practical operational stance: clinicians should not be reassured by the overall rarity of events when evaluating an individual patient with plausible triggers. Instead, the appropriate risk response is rapid recognition and early testing in the right clinical context. In a patient with recent or current SGLT2i exposure, gastrointestinal symptoms, tachypnea, unexplained fatigue, or high anion-gap metabolic acidosis, the pre-test probability of euDKA becomes clinically meaningful regardless of the class-level absolute risk. Consequently, acute-care workflows should prioritize ketone- and acid–base–driven evaluation (serum β-hydroxybutyrate and blood gas/electrolytes) over probabilistic reassurance—particularly in perioperative and ED environments where diagnostic delay is the principal modifiable determinant of morbidity.

## 6. Triggers and High-Risk Phenotypes

Most euDKA episodes occur in the presence of recognizable precipitating factors, and this is one of the most actionable aspects of the condition: triggers are often identifiable in the history and, importantly, many are preventable. In SGLT2i-exposed patients, euDKA typically emerges when the drug’s ketone-permissive pharmacodynamic background intersects with a catabolic state or a reduction in effective insulin activity. Common triggers, therefore, cluster around scenarios that reduce carbohydrate availability, increase counter-regulatory hormones, or limit insulin exposure—most prominently fasting or carbohydrate restriction, dehydration, intercurrent infection, surgery or procedures, insulin dose reduction or omission, and excessive alcohol intake [19]. Rather than acting as isolated “causes,” these triggers usually operate synergistically: for example, gastroenteritis may combine reduced intake, vomiting-driven volume depletion, and missed insulin doses; perioperative pathways may combine fasting, stress hormone surges, and inappropriate withholding of basal insulin.

A central concept is that euDKA is frequently a “relative insulin deficiency” phenomenon. Patients may not be profoundly insulinopenic at baseline, yet the balance between insulin action and counter-regulatory drive becomes insufficient under stress. This is particularly relevant for individuals whose clinical label is T2DM but whose physiology includes limited insulin reserve, such as insulin-treated T2DM, long-standing diabetes with beta-cell exhaustion, or patients with unrecognized autoimmune diabetes/LADA. In these phenotypes, even modest reductions in insulin dosing—sometimes undertaken after SGLT2i initiation due to improved glycemia—can be enough to tip metabolism toward lipolysis and ketogenesis, especially during acute illness [19]. Clinically, this is why euDKA is often reported in patients who appear “stable” from a glycemic perspective but have underlying vulnerability when insulin supply or intake changes.

Dietary patterns are another high-yield trigger domain. Low-carbohydrate diets, ketogenic diets, and prolonged fasting reduce carbohydrate availability and hepatic glycogen stores, thereby increasing reliance on fat oxidation and ketone production; superimposed SGLT2i therapy can further lower insulin tone and amplify this shift [19]. In practice, this means that the “diet history” becomes diagnostic, not merely lifestyle background: recent intentional carbohydrate restriction, weight-loss fasting, or poor intake due to nausea can be the missing link that explains why ketonemia is severe despite near-normal glucose values.

Perioperative medicine deserves special emphasis because it concentrates multiple triggers into a predictable window. Surgical and procedural stress increases counter-regulatory hormones, fasting reduces carbohydrate supply, perioperative nausea/vomiting promotes dehydration, and insulin therapy is often interrupted or inappropriately reduced; together, these factors create a high-risk metabolic environment for euDKA in SGLT2i-treated patients [19]. Indeed, perioperative reports have highlighted that euDKA can present postoperatively with nonspecific symptoms and normal capillary glucose, delaying recognition unless ketone testing is performed early [20,21]. This is particularly relevant for same-day admissions and enhanced recovery pathways, where early discharge and reduced monitoring can intersect with evolving metabolic decompensation.

From an acute-care and prevention standpoint, the practical implication is straightforward: triggers should be treated as a screening tool. In the ED, the combination of recent SGLT2i exposure plus one or more triggers (reduced intake, vomiting/dehydration, infection, perioperative state, insulin interruption, alcohol excess) should lower the threshold for immediate acid–base and ketone assessment even when glucose is not elevated. In prevention, the same trigger map informs “sick-day rules,” perioperative withholding/restart pathways, and clinician education aimed at avoiding insulin over-reduction and ensuring timely ketone testing when symptoms arise [21].

## 7. Clinical Presentation

Clinical presentation of euDKA is frequently nonspecific and therefore prone to under-recognition. Patients commonly report gastrointestinal and constitutional symptoms—nausea, vomiting, abdominal pain, anorexia, malaise, and profound fatigue—often accompanied by dyspnea or a sensation of “air hunger.” On examination, tachycardia and signs of dehydration are common, and respiratory findings may be deceptively unremarkable despite prominent tachypnea. Altered mental status can occur in more severe cases, particularly when acidaemia is marked or when co-triggers such as infection or volume depletion are advanced [22]. The overall clinical picture can resemble a broad range of acute conditions encountered in the ED and perioperative settings, which is precisely why euDKA is often missed early.

A central diagnostic hazard is that glucose values may be normal or only modestly elevated, particularly in SGLT2i-exposed patients. This feature disrupts the typical clinician heuristic that “DKA equals marked hyperglycemia,” and it shifts the presentation toward syndromes that are routinely managed without immediate ketone testing. As a result, euDKA is commonly misattributed to gastroenteritis with dehydration, postoperative ileus, sepsis with hyperventilation, pulmonary embolism work-up pathways, panic/anxiety-related hyperventilation, or adverse drug reactions—especially when vomiting and abdominal pain dominate the initial complaint [22]. In perioperative medicine, euDKA may also be mistaken for expected postoperative nausea, pain-related tachypnea, or early pneumonia/atelectasis, further delaying acid–base evaluation.

Clinically, the most useful bedside clue is respiratory compensation out of proportion to pulmonary findings. Tachypnea (or deep breathing suggestive of Kussmaul physiology) in the absence of clear lung pathology should immediately raise suspicion for a metabolic cause and prompt blood gas assessment. In euDKA, this respiratory pattern can be the earliest overt sign of significant acidaemia, even when hemodynamics are relatively preserved and point-of-care glucose appears reassuring [22]. Similarly, persistent vomiting with dehydration, unexplained abdominal pain, or “sepsis-like” malaise in a patient with recent SGLT2i exposure should lower the threshold for checking serum beta-hydroxybutyrate and electrolytes with an anion gap.

From a workflow standpoint, these bedside observations support a simple operational rule: in any SGLT2i-exposed patient with unexplained tachypnea, gastrointestinal symptoms plus dehydration, or disproportionate malaise, clinicians should prioritize acid–base and ketone assessment early—before anchoring on glucose thresholds. This approach reduces diagnostic delay and allows timely initiation of therapy that targets the underlying ketone-driven process [22].

## 8. Diagnosis in the Emergency Department

Diagnosis of euDKA in the emergency department should be ketone- and acid–base–driven, not glucose-driven. The practical clinical mistake to avoid is treating hyperglycemia as a prerequisite for DKA. In SGLT2i-exposed patients, glycosuria can keep plasma glucose normal or only mildly elevated while ketonemia and acidemia progress; therefore, the operational definition most useful in acute care is high anion-gap metabolic acidosis plus elevated ketones (preferably serum β-hydroxybutyrate), with absent or modest hyperglycemia, in a patient with current or recent SGLT2i exposure. This framing is intentionally pragmatic: it aligns with the pathophysiology (ketone-driven acidosis) and prioritizes the variables that determine immediate management decisions.

A key diagnostic principle is to measure blood β-hydroxybutyrate whenever possible. β-hydroxybutyrate is the predominant ketone body in DKA-spectrum illness, especially in more severe acidemia, whereas urine dipsticks primarily detect acetoacetate and can lag behind clinical evolution and behind therapeutic response [22]. As treatment progresses and the redox state shifts, urine ketones may paradoxically rise or remain positive even while β-hydroxybutyrate falls, making dipsticks unreliable for both diagnosis and monitoring. For ED workflows, this translates into a simple rule: use urine ketones only as a fallback when blood ketone testing is unavailable, and interpret them cautiously in the context of acid–base status.

The minimum ED laboratory panel should be obtained early and ideally in parallel rather than sequentially. A practical “core set” includes venous or arterial blood gas (pH and bicarbonate), electrolytes to calculate the anion gap, serum β-hydroxybutyrate, plasma glucose, creatinine (to gauge renal clearance and dehydration severity), and lactate (to evaluate competing or coexisting causes of high anion-gap acidosis). In many cases, venous blood gas is sufficient for initial decision-making and avoids delays, while repeat assessments should track anion-gap closure and bicarbonate recovery rather than glucose alone.

Interpretation should be structured to prevent anchoring and to identify mixed disorders. High anion-gap metabolic acidosis with elevated β-hydroxybutyrate strongly supports euDKA; however, clinicians should also consider that mixed acid–base disturbances are common in acute illness. For example, vomiting can generate a concurrent metabolic alkalosis that partially masks the severity of ketoacidosis, while respiratory disorders can alter compensation patterns. In this context, the anion gap trend and serial β-hydroxybutyrate measurements are especially valuable because they reflect the underlying ketone burden more directly than a single pH value.

Differential diagnosis is essential, but should be approached in a way that does not delay treatment when euDKA is likely. The differential for high anion-gap metabolic acidosis includes lactic acidosis, uremia/renal failure, toxic alcohol ingestion (ethylene glycol, methanol), salicylate toxicity, and alcoholic or starvation ketosis [23]. Two practical discriminators help at the bedside. First, alcoholic or starvation ketosis typically causes milder acidosis and tends to improve promptly with carbohydrate repletion and hydration alone [23]. Second, lactic acidosis or toxin-related acidosis may explain the anion gap without prominent ketonemia; hence, measuring β-hydroxybutyrate and lactate early allows rapid separation of “ketone-driven” versus “non-ketone-driven” acidosis [23]. Importantly, these conditions can also coexist—sepsis can generate both lactic acidosis and euDKA—so a single competing diagnosis should not prematurely close the case if ketones and acidosis are present.

In practice, euDKA diagnosis is therefore less about a single threshold and more about pattern recognition plus targeted testing. When an SGLT2i-exposed patient presents with unexplained tachypnea, gastrointestinal symptoms with dehydration, perioperative fasting, or intercurrent infection, clinicians should have a low threshold to obtain a blood gas, calculate the anion gap, and measure β-hydroxybutyrate—even if capillary glucose is “reassuring”. This ketone-centric approach shortens time to diagnosis and directly enables timely initiation of fluids, insulin, and electrolyte management, which are most effective when started before severe acidemia evolves (Table 3).

## 9. Management: Protocolized Acute Care

Management of euDKA follows the core principles of standard DKA care but requires a crucial operational adjustment: early dextrose is often needed from the beginning because normoglycemia (or only mild hyperglycemia) is common. The therapeutic goal is not simply to “correct glucose,” but to stop ketogenesis and close the anion gap. This requires sustained insulin exposure to suppress lipolysis and hepatic ketone production, while simultaneously preventing hypoglycemia and avoiding electrolyte complications. As a consequence, the treatment mindset should be explicitly ketone-centric: clinicians should monitor and titrate therapy based on acid–base status and ketone burden rather than on glucose normalization alone [24,25].

Consensus guidelines and major DKA protocols converge on several immediate priorities. First, SGLT2 inhibitors should be discontinued promptly, as ongoing pharmacodynamic effects can perpetuate glycosuria and the ketone-permissive endocrine milieu [24,25]. Second, clinicians should initiate isotonic fluid resuscitation early. Fluids address the pathophysiologic amplifier of euDKA—volume depletion—by restoring effective circulating volume and renal perfusion, which improves clearance of ketones and hydrogen ions and helps attenuate counter-regulatory hormone drive. In practical ED terms, fluids are not merely supportive; they are a disease-modifying intervention that accelerates anion-gap closure when combined with insulin [24,25].

Third, a fixed-rate intravenous insulin infusion is typically required to reliably suppress ketogenesis. In euDKA, insulin is often needed even when glucose is not elevated, which is why dextrose must be added earlier than in classic DKA. The JBDS 2023 guideline explicitly addresses euglycemic DKA and recommends adding 10% dextrose when plasma glucose falls below 14 mmol/L, and considering a reduction in the insulin rate as needed to mitigate hypoglycemia and hypokalemia while maintaining sufficient insulin exposure to switch off ketone production [24]. Conceptually, this is the key management nuance: in euDKA, clinicians frequently “treat the ketones with insulin” while “treating the glucose with dextrose,” so that insulin can continue safely until metabolic resolution.

Electrolyte management—especially potassium—is inseparable from safe insulin therapy. Insulin administration and correction of acidosis shift potassium intracellularly, and hypokalemia increases the risk of arrhythmias and can limit the ability to continue insulin at an effective rate. Therefore, protocols emphasize careful potassium replacement guided by serial measurements and frequent reassessment [24,25]. Clinicians should anticipate that euDKA patients, particularly those with vomiting or diuretic exposure, may present with significant total-body potassium depletion despite normal initial serum values. Ongoing monitoring should include electrolytes, glucose, and acid–base status at intervals consistent with institutional DKA pathways, with the anion gap serving as a practical marker of ketone-driven metabolic recovery.

Bicarbonate therapy remains controversial and is generally reserved for severe acidemia, often cited at pH < 6.9–7.0, and should follow institutional policy and guideline-based thresholds [24,25]. In most cases, the combination of fluids and insulin-driven suppression of ketogenesis will reverse acidosis more safely than bicarbonate, while avoiding potential risks such as paradoxical CNS acidosis and electrolyte shifts.

Metabolic resolution and safe de-escalation require explicit endpoints. Clinically, patients should remain on intravenous insulin (with concurrent dextrose as needed) until anion gap closure and substantial improvement in bicarbonate/pH indicate that ketogenesis has been suppressed and the ketone burden is falling. Transitioning too early risks rebound ketosis, which is particularly relevant in euDKA because glucose values may appear “reassuring” even while ketone clearance is incomplete. When transitioning from intravenous to subcutaneous insulin, protocols recommend an overlap period to maintain continuous insulin coverage and prevent recurrence of ketone production [24,25]. This step is often where failures occur in practice, especially if the patient’s oral intake remains limited or if basal insulin is omitted.

Finally, decisions about restarting SGLT2 inhibitors should be conservative and individualized. SGLT2i should only be restarted after clinical stabilization, restoration of normal oral intake, and resolution of the precipitating trigger. In patients who have experienced euDKA, the risk–benefit balance requires case-by-case reassessment, weighing cardiometabolic benefits against recurrence risk and the feasibility of mitigation strategies (e.g., robust sick-day rules, perioperative pathways, and avoidance of excessive insulin de-escalation). In acute-care workflows, an explicit discharge and follow-up plan—including education on recurrence symptoms and when to seek care—should be considered part of definitive management rather than an optional add-on (Table 4).

## 10. Prevention and Perioperative Management

Prevention of euDKA is particularly high-yield because most episodes arise in predictable, modifiable contexts, and because the clinical harm of euDKA is often driven less by inevitability than by delayed recognition and preventable trigger exposure. In practice, prevention is best framed as a shared responsibility between patients, prescribers, acute-care clinicians, and perioperative systems: the goal is to preserve the substantial cardiorenal benefits of SGLT2 inhibitors while reducing avoidable metabolic decompensation through education, anticipatory planning, and standardized pathways [26].

A cornerstone of prevention is patient-facing education, typically operationalized as “sick-day rules.” Patients should be instructed to temporarily withhold SGLT2 inhibitors during periods of vomiting, reduced oral intake, dehydration, or acute intercurrent illness, and to seek timely medical evaluation if symptoms compatible with ketoacidosis develop—even when home glucose readings appear normal or only mildly elevated [26]. This point is essential: euDKA can evolve precisely because glycosuria blunts hyperglycemia, so a reassuring capillary glucose should not be used as a reason to defer evaluation when nausea, abdominal pain, dyspnea, or profound malaise are present. In higher-risk individuals (e.g., insulin-treated diabetes, prior euDKA, perioperative pathways), education may also include clear instructions on when to check ketones (preferably blood ketones where available) and how to maintain adequate carbohydrate and fluid intake during illness when clinically feasible [26]. Equally important is avoiding inappropriate reductions or omissions of basal insulin during illness, since relative insulin deficiency is a central driver of ketogenesis in SGLT2i-exposed patients [26].

The perioperative setting concentrates multiple euDKA triggers into a narrow time window—fasting, stress hormone surges, variable insulin management, and postoperative nausea or reduced intake—making structured perioperative management essential. For elective surgery, ADA hospital standards recommend stopping SGLT2 inhibitors three days before scheduled procedures (four days for ertugliflozin) [25]. Professional society summaries and perioperative guidance broadly echo these intervals, reinforcing the practical need for scheduled withholding as a default safety strategy within preoperative checklists and medication reconciliation workflows [27]. From an implementation standpoint, these time frames work best when they are embedded into system processes (pre-admission testing protocols, automated medication instructions, and standardized day-of-surgery “hold” lists), rather than relying on ad hoc patient recall or individual clinician awareness.

However, perioperative practice is evolving, and emerging observational data have raised questions about whether a universal “one-size-fits-all” withholding duration is always necessary, highlighting the need for individualized perioperative pathways and prospective evaluation [28]. This does not negate the current pragmatic recommendation to withhold SGLT2i preoperatively; rather, it underscores that perioperative risk is heterogeneous and that institutions may ultimately refine protocols based on procedure type, expected duration of fasting, comorbidity burden, insulin reserve, and the feasibility of postoperative carbohydrate intake and insulin coverage. In the interim, the safest operational stance is to adhere to established withholding guidance for elective procedures while building local pathways that explicitly address monitoring and escalation triggers in the postoperative period [25,27,28].

Prevention does not end at “holding the drug.” A comprehensive perioperative pathway should also specify restart criteria, because premature re-initiation in a patient with ongoing reduced intake, dehydration, or unresolved catabolic stress may recreate the conditions for recurrence. Consistent with acute-care risk framing, SGLT2 inhibitors should generally be restarted only after the patient is clinically stable, euvolemic, eating reliably, and the precipitating trigger has resolved. For higher-risk patients—or those who experienced euDKA—restart decisions should be individualized, balancing the expected benefit against recurrence risk and the patient’s ability to follow prevention measures. Finally, perioperative teams should maintain a low threshold for early ketone testing when postoperative symptoms are disproportionate or atypical: unexplained tachypnea, persistent nausea/vomiting, unexpected fatigue, or high anion-gap metabolic acidosis should prompt immediate evaluation for euDKA regardless of glucose values.

## 11. Special Populations

This review focuses on acute-care pathways, but euDKA is over-represented in surgical, ICU, and cardiorenal populations where SGLT2i are increasingly prescribed, including patients without diabetes [29].

Perioperative risk rises with fasting, dehydration, major surgery, intercurrent illness, and reduced/withheld insulin. Mitigation includes withholding SGLT2i before elective surgery, maintaining basal insulin, avoiding unnecessary fasting, and early blood ketone testing if acidosis or unexplained deterioration develops [30,31].

In emergency surgery or critical illness where drug cessation timing is uncertain, maintain a low threshold for β-hydroxybutyrate measurement and blood gas analysis; titrate therapy to ketone clearance and gap closure rather than glucose alone [32].

Finally, across all populations, prevention and recognition depend on one deceptively simple principle: accurate medication reconciliation. Because SGLT2i pharmacodynamic effects can persist beyond the last dose—particularly ongoing glycosuria and the endocrine–substrate milieu—recent exposure remains clinically relevant even when the patient reports that the drug was stopped “a few days ago.” In acute care, explicitly asking about SGLT2i use within the prior week, recent dose changes, and perioperative withholding/restart timing can be pivotal in correctly framing unexplained high anion-gap acidosis and avoiding diagnostic delay.

## 12. Conclusions

SGLT2 inhibitors have reshaped contemporary cardiometabolic care by delivering meaningful cardiovascular and renal benefits that extend well beyond glycemic control. At the same time, their pharmacology creates a predictable acute-care vulnerability: by promoting glycosuria and altering endocrine–substrate signaling, SGLT2i can lower the threshold for ketogenesis and blunt the classical hyperglycemic warning signal that typically triggers consideration of DKA. The result is euglycemic DKA—a ketone-driven, high anion-gap metabolic acidosis that may present with normal or only mildly elevated glucose and is, therefore, prone to diagnostic delay in emergency and perioperative settings.

For acute-care clinicians, the central operational lesson is simple but essential: DKA is defined by acidosis and ketones, not by glucose alone. In any patient with current or recent SGLT2i exposure, unexplained tachypnea, gastrointestinal symptoms with dehydration, perioperative fasting, infection, or recent insulin interruption should prompt a ketone-first diagnostic approach, including early blood gas assessment, anion gap calculation, and measurement of serum β-hydroxybutyrate. This strategy directly addresses the main modifiable determinant of harm—delayed recognition—while remaining compatible with the broad population-level benefits of SGLT2i therapy.

Management should be protocolized and explicitly targeted at terminating ketogenesis and closing the anion gap. Early isotonic fluids restore perfusion and enhance renal clearance of ketones and acid, intravenous insulin suppresses lipolysis and hepatic ketone production, and early dextrose is frequently required in euDKA to permit ongoing insulin infusion without hypoglycemia. Meticulous electrolyte replacement—particularly potassium—together with frequent reassessment ensures both efficacy and safety until metabolic resolution is achieved. Transition to subcutaneous insulin requires appropriate overlap to prevent rebound ketosis, and decisions about restarting SGLT2i should be individualized after stabilization, recovery of oral intake, and resolution of the precipitating trigger [33,34].

Finally, prevention remains the highest-yield lever for reducing the euDKA burden. Patient education (“sick-day rules”), structured perioperative withholding and restart pathways, and systems-based safeguards (including medication reconciliation and early ketone testing triggers) can substantially reduce missed cases and avoidable events. When these measures are implemented, euDKA becomes a manageable, foreseeable complication—allowing clinicians to preserve the therapeutic value of SGLT2 inhibitors while minimizing preventable morbidity in acute care.

## Figures and Tables

**Table 1:** Sodium–glucose cotransporter (SGLT) inhibitors currently available and their pharmacodynamic characteristics. This table compares SGLT inhibitors in current clinical use, highlighting differences in transporter potency and selectivity (hSGLT2 and hSGLT1 IC_50_ values) and the resulting pharmacodynamic profiles. While selective SGLT2 inhibitors share a class effect driven by renal glucose excretion with osmotic diuresis and transient natriuresis, inter-drug differences in SGLT2/SGLT1 selectivity may influence intestinal SGLT1 engagement and post-prandial glucose handling. These pharmacodynamic features provide the mechanistic background for both therapeutic benefits and context-dependent metabolic adverse events such as euglycemic DKA.

Molecule	Target Profile	hSGLT2 IC50 (nM)	hSGLT1 IC50 (nM)	SGLT1/SGLT2 Selectivity	Key Pharmacodynamic Features
Dapagliflozin	Selective SGLT2 inhibitor	≈1.6	≈803	≈500	Marked renal glucose excretion; transient natriuresis; minimal intestinal SGLT1 inhibition at therapeutic doses.
Empagliflozin	Highly selective SGLT2 inhibitor	≈1.3	≈6278	≈4800	Very high SGLT2 selectivity; predominantly renal mechanism with negligible gastrointestinal SGLT1 effects.
Canagliflozin	SGLT2 inhibitor (lower selectivity)	≈4.4	≈684	≈155	Partial intestinal SGLT1 inhibition at high luminal concentrations; modest effect on post-prandial glucose excursions.
Ertugliflozin	Highly selective SGLT2 inhibitor	≈0.9	≈1960	>2000	Potent SGLT2 inhibition with a predominantly renal pharmacodynamic profile.
Sotagliflozin	Dual SGLT1/SGLT2 inhibitor	≈1.8	≈36	≈20	Combined renal SGLT2 inhibition and intestinal SGLT1 blockade; reduced post-prandial glucose absorption; increased gastrointestinal adverse effects.

Abbreviations: IC_50_, half-maximal inhibitory concentration; SGLT, sodium–glucose cotransporter.

**Table 2:** Euglycemic diabetic ketoacidosis (euDKA): diagnostic criteria and initial work-up. This table summarizes a ketone- and acid–base-driven approach to diagnosing euDKA in the emergency department, emphasizing that normal or mildly elevated plasma glucose does not exclude DKA-spectrum illness. Recommended initial evaluation includes confirmation of ketonemia (preferably serum β-hydroxybutyrate), high anion-gap metabolic acidosis (pH and/or bicarbonate thresholds), calculation and serial monitoring of the anion gap, trigger assessment (including recent SGLT2 inhibitor exposure), and a minimum laboratory panel to support rapid diagnosis and safe protocolized management.

Domain	What to Document	Practical Diagnostic Thresholds/Notes
Glucose	Plasma glucose at presentation (and trend).	euDKA: usually <250 mg/dL (13.9 mmol/L) (can be normal–mildly elevated). Euglycemia does not exclude DKA-spectrum illness.
Ketosis (required)	β-hydroxybutyrate (preferred) or urine ketones.	β-hydroxybutyrate ≥ 3.0 mmol/L strongly supports DKA-spectrum; urine ketones ≥ 2+ can be used if blood ketones are unavailable.
Acidosis (required)	Venous/arterial pH and serum bicarbonate.	pH < 7.30 and/or HCO_3_^−^ < 18 mmol/L. Severity staging by pH/HCO_3_^−^ is useful for triage.
Anion gap	Calculate anion gap: Na^+^ − (Cl^−^ + HCO_3_^−^).	Typically elevated (high anion gap metabolic acidosis). Follow serially until gap closure.
Exclude alternative causes	Lactate, renal function, and toxicology as indicated.	Lactate may be mildly increased, but should not fully explain the anion gap. Assess creatinine/urea; consider osmolal gap/toxins when suspected.
Identify trigger	History + focused exam.	SGLT2 inhibitor exposure, fasting/low-carbohydrate intake, vomiting, infection, surgery, reduced insulin dosing, pregnancy, alcohol use.
Core labs (suggested panel)	Minimum set at presentation.	Glucose, electrolytes, BUN/creatinine, venous blood gas (pH/HCO_3_^−^), β-hydroxybutyrate, CBC ± CRP, urinalysis (ketones/glucose), lactate.

Abbreviations: BUN, blood urea nitrogen; CBC, complete blood count; CRP, C-reactive protein; DKA, diabetic ketoacidosis; euDKA, euglycemic diabetic ketoacidosis; SGLT2, sodium–glucose cotransporter 2.

**Table 3:** Differential diagnosis: euDKA versus classic DKA (and key discriminators). This table contrasts euDKA—often associated with SGLT2 inhibitor exposure—with classic DKA across glycaemia, ketone burden, acid–base profile, glucosuria, and typical triggering contexts, highlighting common diagnostic pitfalls in emergency and perioperative settings and practical tests to separate DKA-spectrum illness from alternative causes of high anion-gap metabolic acidosis.

Feature	euDKA (Often SGLT2-Associated)	Classic DKA	Pitfalls/Discriminators
Glucose	Typically <250 mg/dL (often 100–250).	Typically ≥250 mg/dL (often markedly higher).	Do not rule out DKA-spectrum illness based on glucose alone; assess ketosis and acidosis.
Ketones	Marked ketonemia; β-hydroxybutyrate often ≥3 mmol/L.	Marked ketonemia; β-hydroxybutyrate often ≥3 mmol/L.	Blood β-hydroxybutyrate is preferred; urine ketones can be falsely low early or during recovery.
Acid–base/anion gap	High anion gap metabolic acidosis (pH < 7.30 and/or HCO3^−^ < 18).	High anion gap metabolic acidosis (same thresholds).	Consider mixed disorders (vomiting, diuretics, chronic hypercapnia). Track anion gap closure during treatment.
Urinary glucose	Often disproportionately high for the plasma glucose (drug effect).	Usually parallels the degree of hyperglycemia.	Glucosuria may persist after SGLT2 discontinuation; medication history is essential.
Typical clinical context	SGLT2 inhibitor use; recent surgery/fasting; low-carbohydrate intake; vomiting; infection; reduced insulin; pregnancy; alcohol use.	Missed insulin; infection; myocardial infarction/stroke; pancreatitis; new-onset diabetes.	euDKA can present with ‘normal’ capillary glucose—avoid anchoring bias.
Key alternative diagnoses (ketosis with mild glucose)	Starvation ketosis; alcoholic ketoacidosis.	—	Starvation ketosis usually causes milder acidosis; alcoholic ketoacidosis often has prominent vomiting and a history of alcohol (glucose variable).
Other causes of high anion gap acidosis	Lactic acidosis; uremia/renal failure; toxic alcohols; salicylates.	—	Use lactate, creatinine/BUN, osmolal gap/tox screen when appropriate; these may coexist with DKA and worsen acidosis.

Abbreviations: DKA, diabetic ketoacidosis; euDKA, euglycemic diabetic ketoacidosis; SGLT2i, sodium–glucose cotransporter 2 inhibitor; HCO_3_^−^, bicarbonate; BUN, blood urea nitrogen. Table note: When euDKA is suspected, prioritize confirmation of ketonemia (blood β-hydroxybutyrate) and anion-gap metabolic acidosis over glucose thresholds; consider mixed acid–base disorders and remember that glucosuria may persist after SGLT2i discontinuation.

**Table 4:** Protocolized acute management of euglycemic diabetic ketoacidosis (euDKA) in SGLT2 inhibitor–exposed patients. Stepwise emergency department/ICU workflow summarizing early recognition (anion-gap acidosis plus ketonemia), prompt discontinuation of SGLT2 inhibitors, trigger identification, isotonic fluid resuscitation, intravenous insulin with early dextrose to permit ongoing suppression of ketogenesis, meticulous electrolyte replacement (especially potassium), monitoring until anion-gap closure, and structured transition to subcutaneous insulin with overlap to prevent rebound ketosis, followed by discharge prevention strategies and individualized decisions on SGLT2 inhibitor (re)start.

Phase/Action	What to do (ED/ICU Workflow)	Key Targets/Decision Points	Rationale & Common Pitfalls
(1) Recognize and confirm the phenotype	Treat as DKA-spectrum if high anion-gap metabolic acidosis + ketonemia in a patient with current/recent SGLT2i exposure. Order early: VBG/ABG, electrolytes (anion gap), serum β-hydroxybutyrate, glucose, creatinine/urea, lactate; add toxicology/osmolal gap if indicated.	Do not require marked hyperglycemia. Prefer blood β-hydroxybutyrate over urine ketones. Escalate (ICU/critical care) if severe acidaemia, hemodynamic instability, or refractory electrolyte derangements.	euDKA can be missed when clinicians focus on glucose. Urine dipsticks may lag or mislead during treatment.
(2) Stop SGLT2 inhibitor and identify triggers	Discontinue SGLT2i immediately. Assess triggers in parallel: fasting/low-carb intake, vomiting/dehydration, infection, surgery/procedures, insulin omission or dose reduction, alcohol use.	Document last SGLT2i dose (effects may persist beyond dosing interval). Treat trigger (e.g., sepsis work-up, source control, antiemetics).	Ongoing glycosuria and ketone-permissive milieu may persist after last dose. Failure to address triggers increases relapse risk.
(3) Isotonic fluid resuscitation	Start isotonic crystalloid promptly; reassess frequently. Titrate fluid strategy in heart failure/CKD (smaller boluses, closer monitoring) while maintaining perfusion and urine output.	Targets: improved perfusion, urine output, falling anion gap/β-hydroxybutyrate. Avoid over-resuscitation in congestion-prone patients.	Fluids are disease-modifying: improve renal ketone/H^+^ clearance and blunt counter-regulatory drive. Under-resuscitation delays anion-gap closure.
(4) Intravenous insulin to suppress ketogenesis	Start fixed-rate IV insulin infusion per institutional DKA protocol once potassium is safe. Continue insulin even with normal glucose (goal = stop ketogenesis).	Do not stop insulin solely because glucose is ‘normal’. Hold or delay insulin if severe hypokalemia until corrected (per protocol).	Insulin is the key brake on lipolysis/ketone production. Stopping insulin early → rebound ketosis.
(5) Early dextrose to permit ongoing insulin	Add dextrose earlier than classic DKA; JBDS suggests 10% dextrose when glucose <14 mmol/L. If glucose is already low/normal, start dextrose at initiation to maintain safe glycemia while insulin continues.	Maintain glucose in a safe range while insulin suppresses ketogenesis. Adjust insulin/dextrose to avoid hypoglycemia and rapid K shifts.	In euDKA you ‘treat ketones with insulin’ and ‘treat glucose with dextrose’. Delayed dextrose often forces premature insulin reduction.
(6) Electrolytes (especially potassium) and adjuncts	Check K^+^ frequently; replace aggressively per protocol. Assess Mg^2+^ and phosphate; replace when significantly low or clinically indicated. Continuous ECG monitoring in severe derangements.	Typical protocol logic: if K^+^ is very low, correct before insulin; maintain K^+^ in target range during infusion.	Total-body K depletion is common even with normal initial serum K. Hypokalemia limits insulin therapy and increases arrhythmia risk.
(7) Monitoring and endpoints	Repeat glucose (often hourly) and electrolytes/anion gap/β-hydroxybutyrate at protocol intervals. Track clinical status, urine output, and trigger response.	Primary endpoint: anion gap closure and improving bicarbonate/pH with falling β-hydroxybutyrate. Do not use glucose normalization as the endpoint.	Glucose can look ‘fixed’ while ketones remain high. Mixed acid–base disorders can mask severity—follow anion gap and ketones.
(8) Bicarbonate (selective use)	Reserve for severe acidaemia per institutional policy (often pH < 6.9–7.0).	Use only with close monitoring of K^+^ and ventilation.	Most euDKA resolves with fluids + insulin + dextrose. Bicarbonate can worsen electrolyte shifts if used indiscriminately.
(9) Transition to subcutaneous insulin	Transition only after metabolic resolution and clinical improvement. Provide basal insulin and overlap IV insulin for a protocolized period to prevent rebound ketosis.	Ensure patient is eating/able to tolerate intake. Confirm anion gap closure before stopping infusion.	Premature transition is a common failure point. Basal insulin omission → relapse.
(10) Discharge planning and (re)starting SGLT2i	Provide sick-day rules and clear return precautions. Restart SGLT2i only after stabilization, normal intake, euvolemia, and trigger resolution; individualize after euDKA. Arrange follow-up and document event clearly.	Avoid restarting during ongoing catabolic stress. Reassess risk–benefit in prior euDKA.	Recurrence prevention is part of definitive management. Medication reconciliation errors are common; document last dose and plan.

Abbreviations: ABG, arterial blood gas; CKD, chronic kidney disease; ED, emergency department; euDKA, euglycemic diabetic ketoacidosis; ICU, intensive care unit; IV, intravenous; JBDS, Joint British Diabetes Societies; K^+^, potassium; SGLT2i, sodium–glucose cotransporter 2 inhibitor; VBG, venous blood gas. Table note: Principles are consistent with major adult DKA pathways and JBDS/ADA guidance; follow local institutional protocols for insulin dosing, fluid strategy, monitoring intervals, and electrolyte thresholds.

## Data Availability

No new data were created or analyzed in this study. Data sharing is not applicable to this article.
